# Postradiation trismus in head and neck cancer survivors: a qualitative study of effects on life, rehabilitation, used coping strategies and support from the healthcare system

**DOI:** 10.1007/s00405-024-08609-w

**Published:** 2024-04-08

**Authors:** Susan Aghajanzadeh, Therese Karlsson, Lisa Tuomi, My Engström, Caterina Finizia

**Affiliations:** 1 Department of Otorhinolaryngology, Head and Neck Surgery, Institute of Clinical Sciences, Sahlgrenska Academy, University of Gothenburg, Gothenburg, Sweden; 2 Department of Otorhinolaryngology- Head and Neck Surgery, Region Västra Götaland, Sahlgrenska University Hospital, Gothenburg, Sweden; 3 Institute of Neuroscience and Physiology, Speech and Language Pathology Unit, Sahlgrenska Academy, University of Gothenburg, Gothenburg, Sweden; 4 Institute of Health and Care Sciences, Sahlgrenska Academy, University of Gothenburg, Gothenburg, Sweden; 5 Department of Surgery Gothenburg, Region Västra Götaland, Sahlgrenska University Hospital, 413 45 Gothenburg, Sweden

**Keywords:** Postradiation trismus, Head and neck cancer, Survivorship, Qualitative research, Coping strategies, Pain

## Abstract

**Purpose:**

This study aimed to explore the experiences of head and neck cancer (HNC) survivors with postradiation trismus, specifically how oncological treatment affected their lives, rehabilitation, use of coping strategies, and healthcare experiences. Methods: A qualitative descriptive approach was used and semi-structured interviews of 10 HNC survivors with postradiation trismus were conducted 6–30 months after completing oncological treatment. The interviews were transcribed verbatim and analyzed by qualitative content analysis.

**Results:**

The analysis of interviews yielded four main categories: Bodily symptoms, Effects on life, Support from the healthcare system, and Strategies to handle life and symptoms. Participants reported ongoing problems with xerostomia, dysgeusia, eating, and limited physical fitness. Pain related to trismus was not a major issue in this cohort. Participants expressed limitations in their social lives due to their eating difficulties, yet a sense of thankfulness for life and overall satisfaction with the healthcare they received. Psychological and practical coping strategies developed by the participants were also revealed.

**Conclusion:**

The results highlight areas of unmet need among HNC survivors that healthcare providers can target by establishing multi-professional teams dedicated to individualizing post-cancer rehabilitation care.

**Supplementary Information:**

The online version contains supplementary material available at 10.1007/s00405-024-08609-w.

## Introduction

Survivorship care of patients with head and neck cancer (HNC) is transforming due to the availability of modern radiotherapeutic techniques, which can spare organs at risk [1–3], as well as an epidemiological shift in the disease. Human papilloma virus (HPV)-related HNC cases are increasing, particularly oropharyngeal cancers among patients who are usually younger and have a more favorable prognosis [4]. In Sweden, approximately 1700 patients present with a new HNC diagnosis each year, with a steady yearly increase of 3.2% over the past 13 years [5].

Despite advances in the radiotherapeutic treatment of HNC, survivors experience hardships even after successful curative treatment. Many quantitative studies have been conducted regarding the health-related quality of life (HRQL) of patients with HNC during and after oncologic treatment, indicating that troublesome symptoms tend to persist in the long term [6–9]. Qualitative research regarding HNC survivors’ experiences, coping strategies and symptoms after oncological treatment can highlight unmet care needs and areas of improvement for healthcare providers [10, 11]. This knowledge can, in turn, contribute to HNC survivorship guidelines, including self-management interventions, and care services like speech-language pathology, physical therapy, and nutritional support, among others [12, 13].

Postradiation trismus is a burdensome complication that has been reported in over 40% of HNC survivors at 6 months after completing radiotherapy, and in as many as 28% up to 5 years after completing oncological treatment [6, 14]. In quantitative studies, trismus has been associated with difficulties in eating, performing oral hygiene, speaking, and engaging in intimacy as well as increased facial pain [15, 16]. To our knowledge, no qualitative study aiming to understand the lived experiences, unmet needs, and coping strategies of HNC survivors with postradiation trismus has been conducted. These topics need to be explored in detail to provide effective care and appropriate support to HNC survivors. The aim of this study was to explore the experiences of HNC survivors with postradiation trismus, the potential impacts on their lives, recovery, support needs from the healthcare system and providers, and the use of coping strategies.

## Methods

A qualitative descriptive approach was employed, and semi-structured interviews were carried out using an interview guide (appendix 1). Data were analyzed using qualitative content analysis [17].

### Participants and setting

**Fig. 1:**
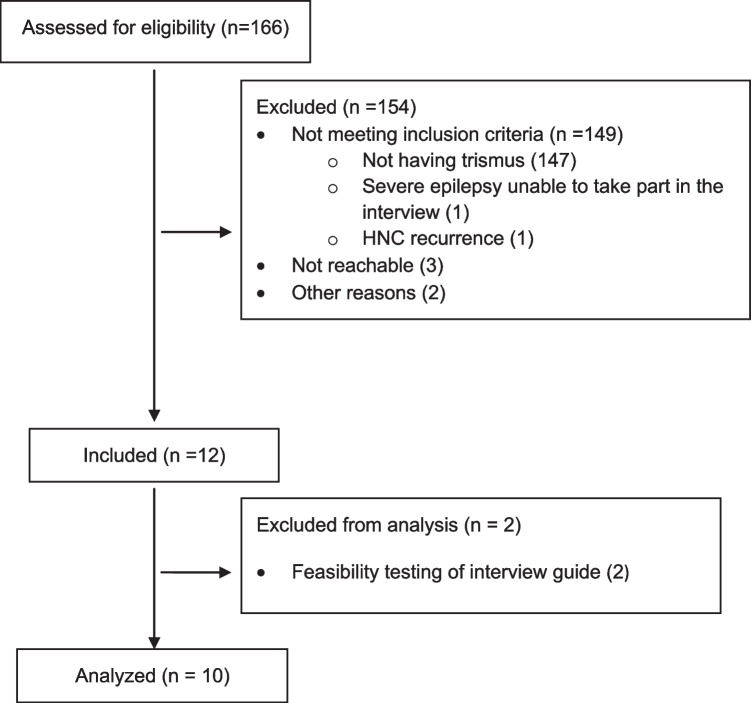
Flowchart showing inclusion process

Between 2021 and 2023, participants were consecutively recruited from a larger quantitative study at the Otorhinolaryngology Department at Sahlgrenska University Hospital in Gothenburg, Sweden that aims to map the relationship between genetic factors and complications after oncological treatment for HNC (Flowchart presented in Fig. 1). The inclusion criteria were age ≥ 18 years, a new HNC diagnosis, receipt of radiotherapy with curative intent with or without chemotherapy or surgery, and a maximal interincisal opening of ≤ 35 mm as proposed by Dijkstra et al. [18]. Exclusion criteria were inability to speak and understand Swedish, a performance status too poor to partake in a lengthy interview or cognitive dysfunction.

Each participant was approached by the first author (SA) by phone, who informed them about the parent study and asked if they were interested in voluntary participation in the current study within the upcoming weeks. Participants were interviewed by the first author via a video meeting, where participants were either at home or another preferred location that offered privacy.

### Data collection

Two pilot interviews were performed to test the feasibility of the interview guide, which resulted in minimal changes in the order of appearance of the questions. Participants were then consecutively interviewed according to the revised interview guide and new interviews were carried out until no new information appeared. After this stage, two more interviews were conducted to ensure saturation. All interviews were audio recorded and transcribed verbatim professionally, using a transcription agency. The mean length of the audio recordings was 43 min (range 20–74 min).

### Data analysis

After achieving saturation, the transcripts were analyzed using manifest qualitative content analysis as described by Graneheim and Lundman [17], in the following steps: (1) each interview transcript was read through several times to achieve an overall understanding of its content, (2) meaning units describing the essence of participants’ experiences were identified and highlighted, (3) meaning units were subsequently condensed without losing its content, (4) these condensed meaning units were labeled with codes, (5) the codes were arranged and grouped into subcategories, (6) subcategories were abstracted into categories representing the manifest content of the data. An example of the analysis process is demonstrated in Table 1.

**Table 1:** Example of the analysis process, moving from meaning unit to category

Meaning unit	Condensed meaning unit	Code	Subcategory	Category
*The mouth opening affects everything really. Even with speaking. Sometimes when I get tired it becomes a bit… Then I must focus when I talk so that it won’t sound slurred*	Trismus causes speaking difficulties, especially when tired	Speaking difficulties due to trismus	Problems related to eating and trismus	Bodily symptoms

Authors (SA) and (ME) initially analyzed the transcripts separately. The preliminary sub-categories and categories were then reflected over and discussed with all the authors until consensus was achieved.

### Ethical considerations

Patients received both verbal and written information before participating in the study. Informed written consent was provided by all participants. Patients were informed that study participation was voluntary and their decision to take part or not would not have any impact on their continuing care. They were also informed on two occasions about the right to withdraw at any time without having to specify the reason. Participants were informed that they could receive follow-up support if the interview elicited feelings of discomfort or distress. During the interviews, the interviewer was attentive to signs of discomfort or signals of wishing to withdraw from the interview. During the writing process, efforts were made not to provide specific information about the participants that could risk revealing their identities. No compensation was offered and none of the authors had a healthcare provider role for any of the participants. All collected information was treated according to the European General Data Protection Regulation (GDPR).

## Results

**Fig. 2:**
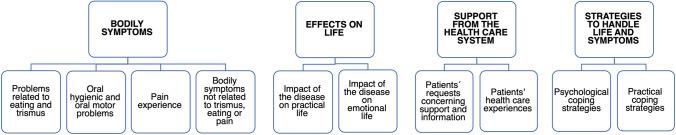
Flowchart description of the four categories and ten subcategories obtained from the data analysis

In total, 10 participants were interviewed, and their characteristics are presented in Table 2. The analysis yielded four categories: (1) bodily symptoms, (2) effects on life, (3) support from the healthcare system, and (4) strategies to handle life and symptoms. These categories were based on ten subcategories, as demonstrated in Fig. 2.

**Table 2:** Participant characteristics

	n (%)
Age, Mean (Range)	68 (56–77)
Sex	
Male	5 (50)
Female	5 (50)
Living alone	
Yes	3 (30)
No	7 (70)
Educational level	
Primary	2 (20)
Secondary	4 (40)
University	4 (40)
Employment status	
Working	4 (40)
Sick leave	0 (0)
Retired	6 (60)
Cancer stage	
I	2 (20)
II	3 (30)
III	3 (30)
IV	2 (20)
Tumor site	
Oral cavity	2 (20)
Oropharynx	8 (80)
Cancer treatment	
RT only	0 (0)
Surgery + CRT	1 (10)
Surgery + RT	1 (10)
CRT	8 (80)
Time since treatment completion (months)	
8–< 12	7 (70)
12–< 30	2 (20)
= 30	1 (10)
MIO at time of interview (mm)	
13–< 31	5 (50)
31–≤ 35	5 (50)

*CRT* chemoradiotherapy, *RT* radiotherapy, *MIO* maximal interincisal opening

### Category 1.

*Bodily symptoms* was created from the subcategories of *Problems related to eating and trismus*, *Oral hygienic and motor problems*, *Pain experience,* and *Bodily symptoms not related to trismus, eating or pain*.

#### Problems related to eating and trismus

Study participants mainly expressed having difficulties related to eating and hyper- or hypoproduction of saliva. Xerostomia was an ongoing complaint for at least four participants. Dysgeusia in the post-treatment period was a recurring issue with a variable symptom description. For instance, one participant avoided sweet foods because of the loss of taste for sweetness, while another could taste sweetness the best and preferred sweet foods. A recurring problem among participants almost a year after treatment completion was the inability to drink wine because of feeling a burning sensation in the mouth or a severe sensation of dryness whereas beer was described by some to be more pleasant to drink. For one participant, eating difficulties were also caused by dental loss. The affected participant was still awaiting dental treatment at the time of the interview (13 months post-treatment). Trismus caused problems with speaking, eating, and kissing. One participant, who was interviewed 8 months after finishing combined surgical and radiotherapeutic treatment, suffered from trismus so severe that he/she could only eat mashed foods and avoided meat completely. However, for the remaining participants, trismus seemed to be a complication that was better tolerated than dysgeusia and dryness of the mouth.

*“If I want to eat a hamburger or bite an apple, that’s when it’s the most burdensome so to say. But other than that, I don’t really suffer from it.”* (Participant 4)

*“I must say that what affects me the most is… the taste changes that occur in the mouth. Taste and dryness and these kinds of things. It’s what I think I’ve been mostly affected by. Even if it’s getting better.”* (Participant 10)

#### Oral hygienic and motor problems

Participants mostly described burdensome sialorrhea during the initial period after oncologic treatment. Some participants had problems with phlegm production during convalescence, while others complained of a specific spot in their mouth where different foods got stuck. For one participant, another lingering issue was the loss of sensation in the corner of their mouth, which caused difficulties in knowing when they salivated visibly, or when foods became stuck onto their skin. The feeling of tongue fatigue or tongue swelling was also mentioned, as well as the tendency to accidentally bite one’s lip due to decreased sensation.

*“I keep having to clear my throat all the time… Some days I spit all day”. *(Participant 2)

*“Sometimes my wife has to let me know, since I can’t feel anything on this side… So food can sit there without me noticing and then she’ll tell me.”* (Participant 8)

#### Pain experience

Regardless of the range of maximal interincisal opening, cancer type or stage, or treatment modality, pain related to trismus was not a major issue for the participants. For example, one participant did not experience pain after the tumor was gone. The pain described was mostly intermittent, short-lasting, or associated with chewing or yawning.

*“Yes, a little bit of a headache up here where the jaw attaches by the ear. It can hurt here sometimes. At times when I eat, it’s like a knife stab there. But it’s not a constant pain, it’s temporary and goes away after half a minute.”* (Participant 8)

*“I used to think it was pleasant to yawn. But now I don’t find it pleasant because there is a resistance and it’s slightly painful… It’s not really painful but rather uncomfortable.”* (Participant 9)

#### Bodily symptoms not related to trismus, eating or pain

Limited physical fitness, muscle weakness and fatigue were mentioned as residual symptoms by some participants, as well as mild changes in voice quality. One participant complained of lymphoedema of the anterior neck. A few participants felt like they were almost back to the same physical fitness that they were in before the disease.

*“I’ve been outside walking a lot during the whole time, just to keep the body moving. So, I haven’t lost a lot of my fitness. I lost five kilograms, and I needed it so to say”* (Participant 4)

### Category 2.

*Effects on life* was created from the subcategories of *Impact of the disease on practical life* and *Impact of the disease on emotional life*.

#### Impact of the disease on practical life

The impact of the disease and treatment on life varied considerably across the interviewed participants’ reports. For some participants, impacts were limited to their eating difficulties, which turned mealtime with friends or family into a negative experience, mainly because the mealtime was now considerably longer. For others, however, it was not a problem. Some participants mentioned that their situation was constantly improving. One participant felt largely limited because of an open intraoral wound, which caused salivary leakage. One participant did not go back to the office during the period he had a nasogastric tube. Another participant was particularly impacted by the length of period after treatment during which complications continued to arise, which led them to cancel a planned trip.

*“I have considered this year to be a lost year for me. I haven’t done anything, you know.”* (Participant 2)

*“I can’t eat whatever I would like since I can’t chew. And as I said, it burns… some things burn like fire inside the mouth but it’s… It’s nothing that stops me from joining others to eat.”* (Participant 3)

*“It inhibits me from going out and eating at restaurants and spending time with friends. Because it takes me so long. So that’s what I feel is difficult.”* (Participant 5)

*“At least on the weekend I usually cook for my family which is fun, even if I can’t sense taste really well, I still manage to succeed mostly.”* (Participant 1)

#### Impact of the disease on emotional life

In this subcategory, the study participants presented a variety of ways they had been affected emotionally by HNC and its treatment. Some experienced unaltered psychosocial conditions. Others wished to avoid new contacts while still wanting to be social. Some participants reported a lack of desire for physical intimacy. Many participants described emotional support provided by family, friends, or neighbors. Fear of recurrence of their cancer was mentioned by a few participants. Some also mentioned that the disease reminded them that life is delicate, and they now appreciated the little things in life more.

*” I’m grateful and positive to life and enjoy every morning when I can put my feet on the floor”* (Participant 6)

*“I feel very fragile so to say. It’s easier for me to both laugh and to cry now. I become very emotional for others if anything happens.”* (Participant 8)

### Category 3.

*Support from the healthcare system* was created from the subcategories of *Patients´ requests concerning support and information* and *Patients’ healthcare experiences.*

#### Patients’ requests concerning support and information

Participants mostly expressed being well-informed about the disease, its treatment, and possible complications that may occur. One participant wished for a brochure with a timeline of when to expect specific side effects or complications after the treatment and the length of time until resolution. Another expressed that knowing more about everything that may occur would not necessarily be helpful. Others wanted to have information about support groups of HNC survivors early on, from the start of radiation therapy. One participant asked for an outpatient clinic that addressed altered taste function after oncological treatment. Other wishes were to be offered counseling and facilitation when ordering subsidized vehicular transports to and from the hospital.

*“I don’t think it would have helped. It’s so difficult to imagine how things will turn out. Even if they had said that you will probably not be able to open your mouth more than 2,5 centimeters in a year, I don’t think I would have understood it properly.”* (Participant 8)

*“It was the cancer… It was a serious disease so to say. So that’s what I concentrated on. Mouth opening difficulties, that’s secondary, so I didn’t think about it a lot. But the information I received was good.”* (Participant 3)

#### Patients’ healthcare experiences

In general, participants were content with the healthcare they had received, and many felt looked after by their healthcare providers. A few participants expressed disappointment with how long it took for their primary care practitioner to raise suspicion of HNC and to receive a subsequent referral to an otorhinolaryngologist. Others were disappointed with not receiving their follow-up visits and radiology appointments in time. The contact nurse function at the responsible ENT clinics was appreciated, as well as counseling before and after treatments, and dietitian and dental follow-ups. One participant mentioned that their contact nurse had called them regularly to ask how they were doing. Another expressed that the contact nurse helped them get in touch with other healthcare providers when they needed it. One participant was especially thankful for the treatment and availability of the radiation oncology unit at the University Hospital.

*“And the personnel from Radiation Oncology were amazing. I was always in the same radiation room, so I got to know them. They were so pleasant and encouraging.”* (Participant 10)

### Category 4.

*Strategies to handle life and symptoms* was created from the subcategories of *Psychological coping strategies* and *Practical coping strategies.*

#### Psychological coping strategies

Participants reported various psychological coping mechanisms. A few mentioned having a positive attitude that was thought to favor perseverance and which helped them cope with the fear of cancer recurrence. Other psychological coping mechanisms were acceptance of their situation, in general, and adapting to life and living with trismus. Participants also mentioned that the bodily symptoms remaining from the cancer and its treatment felt less important as long as the cancer itself was cured.

*“I don’t think about having had cancer so much, I mean it existed and now it seems fine so I’m going to have to think that it will continue that way for a while.”* (Participant 3)

#### Practical coping strategies

Participants reported a variety of practical strategies to help cope with the experience of receiving a cancer diagnosis and going through treatment, as well as specific strategies to cope with the remaining symptoms. For example, participants described reading books helped them significantly during nights when they could not sleep while receiving oncological treatment, as well as in the survivorship period to help clear their minds. They also reported that the sense of meaning and purpose they felt while working was a source of solace for them. Spending time with grandchildren was a recurring coping strategy. A few participants received help with coping strategies through conversational therapy with a hospital counselor. Other strategies mentioned were medical yoga with a focus on breathing techniques and having a measuring tape that they cut off a piece from every time they went through a radiotherapy session. Watching the tape shrink in size was a helpful reminder that the goal of treatment completion was approaching.

*“I tried to go back to work as early as possible, but it was during the pandemic, so I could work from home. I felt that it was good for me because it helped to remove focus away from my disease.”* (Participant 5)

Several practical strategies were reported regarding eating problems. For instance, participants described that eating everything, even though it was difficult or didn’t taste normal, helped them cope with their situation. One participant reported eating the same foods as his/her spouse but finely cutting everything into small pieces instead of omitting foods that were difficult to eat. Sparkling water was helpful to some participants with swallowing difficulties as was substituting beef with vegetarian meat-like alternatives. The need to use liquids to facilitate meals was also reported.

*“It’s difficult to swallow and I have to use a lot of liquids for meals and push foods in between my teeth. But it works.”* (Participant 2)

One participant demonstrated a technique to increase their salivary production to help with xerostomia where they made repetitive movements with their lower jaw from side to side. Another participant used paracetamol regularly to help alleviate trismus.

*“I sense a swelling which inhibits me. It worsens my mouth opening ability, so I take paracetamol for it to alleviate a bit.”* (Participant 5)

Furthermore, participants explored several jaw exercise strategies. Three participants avoided jaw trainers completely and only used their fingers. This was either due to the jaw trainer causing pain or due to it feeling too bulky to use. One participant reported performing fewer jaw exercises due to an improvement in trismus-related symptoms over time and another stopped exercising after reaching a certain level of maximal interincisal opening.

*“I trained multiple times daily during a few weeks or months, I don’t recall exactly, and then more sporadically. You forget about it when you have less problems with mouth opening. That’s just how it is.”* (Participant 3)

## Discussion

This study is, to the best of our knowledge, the first qualitative study focusing specifically on HNC survivors with postradiation trismus and aimed to detail their experiences, coping strategies, and healthcare supports. The participants in the study reported a range of impacts that the disease and its treatment had on their lives. Our results showed that patients experienced various degrees of problems with eating, altered taste, and xerostomia. This is in line with previous research on symptom burden concerns in patients with HNC after oncologic treatment. Crowder et al. (2019) interviewed 31 HNC survivors who were disease-free for at least 6 months and up to 9 years. The most common symptoms were dysphagia, xerostomia, taste alterations, and bothered chewing [19]. In an observational longitudinal cohort study, patients with HNC reported the highest symptom burden from dry mouth as well as sticky saliva. In the same study, eating limitations were significantly more prevalent among HNC patients with postradiation trismus compared to those without trismus [6].

While xerostomia was a common complaint among the study participants, sialorrhea was also mentioned as a burdensome symptom during the initial period after receiving radiation therapy. This is contradictory to the xerostomia effect and, as Bomeli et al. (2012) noted, a less frequent, but troublesome complication of HNC treatment. Sialorrhea often occurs as a manifestation of radiation-related dysphagia [20]. Thick secretions can develop due to radiotherapy and last for weeks and up to 6 months post-treatment. Swallowing problems can then result in the pooling of secretions, with increased potential for aspiration [21]. One participant who had completed chemoradiotherapy for tonsillar cancer 9 months prior to the interview described tongue fatigue and a sensation of swelling. This type of symptom is not specifically asked about in widely-used HNC HRQL questionnaires [22, 23] and therefore seems to be infrequently reported. Decreased range of motion of the tongue due to fibrosis or damaged nerve innervation has, however, been reported after chemoradiotherapy [24], which could potentially explain this participant’s complaints.

Generally, study participants had an overall positive experience with the healthcare they received. However, some unmet needs were identified, which are similar to the results of previous qualitative studies. Crowder et al. (2021) reported that survivors did not know how long treatment-related consequences would persist and that many were hoping to return to normal, or as they were before the malignancy [12]. In our study, one participant strongly wished for specific information since he/she had expected a faster return to “normal.” Instead, new symptoms kept emerging, which affected the participant negatively over one year. In their qualitative study of HNC survivors, Saghafi et al. (2023) noted that participants were poorly informed of the severe nature of the treatment and lacked information about treatment-related symptoms and long-lasting side effects [10]. Likewise, Oswald et al. (2022) reported that HNC survivors need specific interventions, including education about acute and long-term side effects and symptom management [25]. These findings indicate that the information patients receive may need to be improved and tailored to address specific needs. Traditionally, physicians inform patients about possible side effects and long-term effects before they commence treatment. However, according to the narrative report by Kessels, a patient may not be perceptive to a lot of information soon after a new cancer diagnosis [26]. Healthcare providers could thus in some cases consider repeating this information after the treatment has been administered. Nevertheless, healthcare providers also need to ensure that patients are informed about the specific supports and services their healthcare team can provide for them.

Overall, the participants felt they had come a long way in terms of self-care by developing practical and psychological coping strategies. An interesting psychological strategy was the “downplaying” of symptoms, particularly in the presence of disease-free survival. The “downplaying” phenomenon among patients with HNC was first described by Wells, where a perception that others were worse off seemed to prevent acknowledgment of one’s own difficulties [27]. In their qualitative study, Swore Fletcher et al. reported that many HNC survivors described themselves as “lucky” due to factors such as treatment success, excellent care, or family support, among others [28]. Our results also showed that participants had developed different coping mechanisms related to eating such as using more liquids or avoiding wine because of feeling a burning sensation or harsh taste. These findings echo the complaints and coping strategies identified in past works. Einarsson et al. interviewed patients up to 2 years after completing treatment for HNC and found reports of difficulties such as the inability to drink wine or eat burgers. Patients had also developed strategies, such as using liquids to facilitate swallowing as well as taking smaller bites of food [29].

The collective findings highlight areas within the HNC survivorship care that require improvement. While a patient is often asked about symptoms of the disease and its treatment, their questions regarding unmet healthcare needs and specific emotional support are less commonly explored. The study also confirms the importance of a multi-professional approach to HNC survivorship care after oncologic treatment, and a focus on individualized HNC specific rehabilitation, preferably via specific rehab teams. In this approach, contact nurses serve an important function by providing consistent support for the patient and aid in developing a rehabilitation plan, which the Swedish National Board of Health and Welfare recommends be made available for all cancer survivors [30]. Recommendations for HNC survivorship care have been developed by the European Head and Neck Society. These recommendations focus on the information and supportive care needs of HNC survivors related to cancer and its treatment, adverse effects, speech pathology, and other health concerns, as well as available support services. They also provide or refer HNC survivors to appropriate resources to meet identified needs [31]. Healthcare teams may also want to consider identifying patients who may benefit from self-management interventions, as they can be an efficient strategy for helping cancer survivors in their recovery journey. These interventions can help empower the survivors through enhanced knowledge and by gaining support from fellow survivors. More insight is, however, needed into how to best design and deliver specific interventions to HNC survivors [32].

Lastly, pain was not a major problem among the interviewed patients, which was surprising since earlier research indicates the opposite trend. For example, up to 5 years following radiotherapy for HNC, 41–54% of survivors reported facial pain, with a higher prevalence at 3 months post-radiotherapy (70%) [16, 33]. Patients in these studies were treated between 2007 and 2012, and no interviews were carried out, while the current cohort was included between 2020 and 2022. Participants in this study were thus receiving newer radiation techniques, i.e., Volumetric Modulated Arc Therapy (VMAT) in comparison to Intensity-Modulated Radiation Therapy (IMRT) and even older techniques. Newer techniques can reduce the dose delivered to normal tissue and organs at risk [3], which could be one explanation behind our cohort reporting less pain. However, this is highly speculative since there is insufficient data on HRQL and pain among HNC patients receiving different treatment modalities. In a recent qualitative study of 10 HNC survivors, oral pain and pain related to the jaw area were more commonly reported [10] than in the current study, which could be because six out of ten of these survivors had undergone combination treatment, including surgery. Lastly, when trismus occurred, all participants in our cohort received some sort of jaw exercise program, either with a jaw training device or manually, with varying frequency and length. This could also partly explain the lower prevalence of pain among the cohort, since jaw exercises have demonstrated positive effects on mouth opening and pain up to 3 years following oncologic treatment [33–35].

This study yielded some valuable insights that can be further explored. The participants were able to express their experiences as well as met and unmet needs freely, which is a strength of qualitative methodology. Furthermore, the manifest analysis process allowed the participants’ statements to be expressed in the data with minor interpretation from the researchers. The time span during which the interviews were conducted was broad enough to explore experiences both early and late after treatment completion. Furthermore, all interviews were performed by the same researcher. One methodological consideration of this study is the limited number of participants. However, the resulting content was rich and the material was perceived as saturated by the final set of interviews. One improvement that could be considered for future studies is to interview the same participant over time to detect changes in their survivorship experiences and how their needs evolve. As some practical coping strategies seem to recur across studies, a systematic review assessing the quality of evidence should be performed and relevant results be added to HNC care guidelines.

## Conclusions

The results showed that HNC survivors with trismus continued to report bothersome symptoms for up to 2 years after oncological treatment, but that they developed coping strategies to better handle life after treatment and manage the impacts of symptoms. They also expressed some unmet healthcare needs and expectations, including the need for further information about long-lasting symptoms and improved follow-up care. In light of these findings, healthcare providers should work towards establishing multi-professional teams dedicated to providing individualized post-treatment rehabilitation care for HNC survivors.

### Supplementary Information

Below is the link to the electronic supplementary material.

Supplementary file1 (DOCX 24 KB)

Supplementary file2 (DOCX 20 KB)

## Data Availability

The data supporting the findings of this study are available from the corresponding author, upon reasonable request.
