# Supplementary material for: Postradiation trismus in head and neck cancer survivors: a qualitative study of effects on life, rehabilitation, used coping strategies and support from the healthcare system
Source: Eur Arch Otorhinolaryngol. 2024 Apr 8;281(7):3717–26. doi: 10.1007/s00405-024-08609-w (PMC11211121; doi:10.1007/s00405-024-08609-w)
Supplement: Supplementary file 1 — Supplementary file1 (DOCX 24 KB) [file 405_2024_8609_MOESM1_ESM.docx]

**Consolidated criteria for reporting qualitative studies (COREQ): 32-item checklist**

***Postradiation trismus in head and neck cancer survivors- a qualitative study of effects on life, rehabilitation, used coping strategies and support from the healthcare system***

| **No .** | **Item .** | **Guide questions/description** | **Authors answer, line in manuscript when applicable** |
| --- | --- | --- | --- |
| **Domain 1: Research team and reflexivity** |  |  |  |
| Personal Characteristics |  |  |  |
| 1. | Interviewer/facilitator | Which author/s conducted the interview or focus group? | Susan Aghajanzadeh  ***Page 2*** |
| 2. | Credentials | What were the researcher's credentials? *E.g. PhD, MD* | **SA:** MD  **TK:** Associate professor, PhD  **LT:** Associate professor, PhD  **ME:** Associate professor, PhD  **CF**: Professor, PhD |
| 3. | Occupation | What was their occupation at the time of the study? | **SA:** MD  **TK**: MD  **LT**: Senior lecturer, SLP  **ME:** Senior lecturer, CNS  **CF**: MD, University Hospital Research Director |
| 4. | Gender | Was the researcher male or female? | **SA:** Female  **TK**: Female  **LT**: Female  **ME:** Female  **CF**: Female |
| 5. | Experience and training | What experience or training did the researcher have? | **ME, LT, TK & CF;** Senior researchers (associate professor/professor) and experienced research supervisors)  **SA:** Active researcher, MD |
| Relationship with participants |  |  |  |
| 6. | Relationship established | Was a relationship established prior to study commencement? | No relationship beyond contact regarding inclusion and the performed interviews by SA.  ***Page: 3*** |
| 7. | Participant knowledge of the interviewer | What did the participants know about the researcher? e*.g. personal goals, reasons for doing the research* | All patients received both verbal and written information as well as signed an informed consent about the study including: Reason for conducting the study, why they were asked to participate, aim, what it would mean to them if they agreed to participate, confidentiality, how the result would be presented and that they could withdraw their consent at any time. |
| 8. | Interviewer characteristics | What characteristics were reported about the interviewer/facilitator? e.g. *Bias, assumptions, reasons and interests in the research topic* | **SA** is a consultant otorhinolaryngologist but has not had a health providing role with any participants. |
| **Domain 2: study design** |  |  |  |
| Theoretical framework |  |  |  |
| 9. | Methodological orientation and Theory | What methodological orientation was stated to underpin the study? *e.g. grounded theory, discourse analysis, ethnography, phenomenology, content analysis* | A manifest content analysis was used in accordance with Graneheim & Lundman*  *Graneheim UH & Lundman B (2004): Qualitative content analysis in nursing research: concepts, procedures and measures to achieve trustworthiness. Nurse Educ Today 24, 105-112.  ***Page: 3*** |
| Participant selection |  |  |  |
| 10. | Sampling | How were participants selected? *e.g. purposive, convenience, consecutive, snowball* | Consecutive  ***Page: 2*** |
| 11. | Method of approach | How were participants approached? e*.g. face-to-face, telephone, mail, email* | By phone  ***Page****:* ***2*** |
| 12. | Sample size | How many participants were in the study? | 10  ***Page: 3*** |
| 13. | Non-participation | How many people refused to participate or dropped out? Reasons? | Two.  One patient declined participation because of epilepsy and inability to participate in video meetings. One patients declined because of recurrent head and neck cancer. |
| Setting |  |  |  |
| 14. | Setting of data collection | Where was the data collected? e*.g. home, clinic, workplace* | Via video meeting (home, office)  ***Page: 2*** |
| 15. | Presence of non-participants | Was anyone else present besides the participants and researchers? | No. |
| 16. | Description of sample | What are the important characteristics of the sample? *e.g. demographic data, date* | Demographic data presented in Table 2. |
| Data collection |  |  |  |
| 17. | Interview guide | Were questions, prompts, guides provided by the authors? Was it pilot tested? | A semi-structured interview guide served as a script to ensure that all participants received the same questions.  Two pilot interviews were conducted to ensure that the questions in the interview guide addressed the study aims; thereafter an additional question was added to the interview guide.  ***Appendix 1 and Page: 2*** |
| 18. | Repeat interviews | Were repeat interviews carried out? If yes, how many? | No. |
| 19. | Audio/visual recording | Did the research use audio or visual recording to collect the data? | All interviews were audio recorded.  ***Page: 2*** |
| 20. | Field notes | Were field notes made during and/or after the interview or focus group? | Not as a standard procedure. |
| 21. | Duration | What was the duration of the interviews or focus group? | Mean interview time was 43 minutes (range: 20-74 min).  ***Page: 2*** |
| 22. | Data saturation | Was data saturation discussed? | Yes  ***Page: 2*** |
| 23. | Transcripts returned | Were transcripts returned to participants for comment and/or correction? | No. |
| **Domain 3: analysis and findings** |  |  |  |
| Data analysis |  |  |  |
| 24. | Number of data coders | How many data coders coded the data? | Two. **SA** and **ME**. |
| 25. | Description of the coding tree | Did authors provide a description of the coding tree? | Yes. |
| 26. | Derivation of themes | Were themes identified in advance or derived from the data? | Derived from data.  ***Page: 3*** |
| 27. | Software | What software, if applicable, was used to manage the data? | Word and Excel (Microsoft Office) |
| 28. | Participant checking | Did participants provide feedback on the findings? | They will receive the published manuscript upon request. |
| 29. | Quotations presented | Were participant quotations presented to illustrate the themes / findings? Was each quotation identified? e*.g. participant number* | Yes, identified with participant number.  ***Page: 4-10*** |
| 30. | Data and findings consistent | Was there consistency between the data presented and the findings? | Yes. |
| 31. | Clarity of major themes | Were major themes clearly presented in the findings? | Yes.  ***For example see pages 4-10*** |
| 32. | Clarity of minor themes | Is there a description of diverse cases or discussion of minor themes? | Diverse cases are most often presented as: *”A few participants …”* or *“One participant…”.*  ***For example see pages 4-10*** |
