# Supplementary material for: Postradiation trismus in head and neck cancer survivors: a qualitative study of effects on life, rehabilitation, used coping strategies and support from the healthcare system
Source: Eur Arch Otorhinolaryngol. 2024 Apr 8;281(7):3717–26. doi: 10.1007/s00405-024-08609-w (PMC11211121; doi:10.1007/s00405-024-08609-w)
Supplement: Supplementary file 2 — Supplementary file2 (DOCX 20 KB) [file 405_2024_8609_MOESM2_ESM.docx]

**Interview guide: 6 to 24 months after completion of treatment**

**Perception of the impact of trismus on life, recovery and coping strategies**

1. I would like you to start by telling me if and how you feel that trismus i.e. mouth opening difficulties affect your everyday life?

2. How do you feel that the treatment you have undergone because of the tumor has affected your everyday life?

2.1 How do you feel about your ability to manage your everyday life after the treatment?

2.2 Did you feel that you got the support you needed to be able to return to

activities that are meaningful to you (work, friends, acquaintances)?

3. Can you tell us more about physical recovery? For example, eating/drinking, being

physically active in your daily life?

*3.1 In general*

*3.2 Focus on trismus (Trained with jaw training device? Tell me about it).*

4. Can you tell me more about the psychosocial experience after completing treatment? For example, your ability to socialize and be social with family, friends, colleagues.

*4.1 In general*

*4.2 Focus on trismus*

5. Can you tell me more about how your emotional life has been affected by the treatment?

*5.1 In general*

*5.2 Focus on trismus*

6. Is there any experience that you have gained regarding trismus that you feel can help you better manage other side effects/complications after treatment?

7. Do you have any strategies that help you cope with other difficulties in everyday life caused by your treatment?

*7.1 In general*

*7.2 Focus on trismus*

8. Have you recently been on sick leave?

**Pain**

9. Do you have pain due to trismus?

9.1 How do you feel about your own ability to self-manage your pain caused by trismus after surgery?

9.2 For example, do you use any strategies to manage pain?

10. Do you feel that you received the information and support you needed to manage the pain caused by trismus in connection with your return visits to the clinic after treatment?

11. Have you experienced participation in the pain treatment?

11.1 If so, in what way?

12. Have you communicated about your pain due to trismus to healthcare professionals?

12.1 If so, what have you discussed?

13. Has any instrument been used to assess your pain?

**Support from healthcare system**

14. Prior to your treatment, for example during the information session with your physician, were you informed that you could suffer from trismus after your completion of treatment?

14.1 Did you feel that you received enough information about this?

14.2 If not, what more information would you have liked?

15. If we focus on the communication between you and the healthcare professionals in relation to your treatment and follow-up, could you tell us more about it?

15.1 For example, did you feel seen and listened to? Did you get the support you needed?

16. Is there anything you missed that you feel could have helped you to better manage trismus and the pain that can occur after treatment?

17. Is there any other health care support that you missed that is not directly related to

trismus?

18. Is there anything else you think I have failed to mention that has been important to you?

**European Archives of Oto-Rhino-Laryngology**

**Postradiation trismus in head and neck cancer survivors- a qualitative study of effects on life, rehabilitation, used coping strategies and support from the healthcare system**

Susan Aghajanzadeh MD ^1,2^ Therese Karlsson MD, PhD ^1,2^ Lisa Tuomi PhD ^1,3^ *My Engström, PhD ^4,5^ *Caterina Finizia MD, PhD ^1,2^

^1^ Department of Otorhinolaryngology, Head and Neck Surgery, Institute of Clinical Sciences, Sahlgrenska Academy, University of Gothenburg, Gothenburg, Sweden

^2^ Region Västra Götaland, Sahlgrenska University Hospital, Department of Otorhinolaryngology- Head & Neck Surgery, Gothenburg, Sweden

^3^ Institute of Neuroscience and Physiology, Speech and Language Pathology Unit, Sahlgrenska Academy, University of Gothenburg, Gothenburg, Sweden

^4^ Institute of Health and Care Sciences, Sahlgrenska Academy, University of Gothenburg, Gothenburg, Sweden

^5^ Department of Surgery Gothenburg, Region Västra Götaland, Sahlgrenska University Hospital, Gothenburg, Sweden

*Equal contributions

Corresponding author: My Engström, Sahlgrenska University Hospital, SE-413 45 Gothenburg, Sweden; E-mail: my.engstrom@gu.se
